# Fingolimod after a first unilateral episode of acute optic neuritis (MOVING) – preliminary results from a randomized, rater-blind, active-controlled, phase 2 trial

**DOI:** 10.1186/s12883-020-01645-z

**Published:** 2020-03-03

**Authors:** Christian Albert, Janine Mikolajczak, Anja Liekfeld, Sophie K. Piper, Michael Scheel, Hanna G. Zimmermann, Claus Nowak, Jan Dörr, Judith Bellmann-Strobl, Claudia Chien, Alexander U. Brandt, Friedemann Paul, Olaf Hoffmann

**Affiliations:** 1 Department of Neurology, Alexianer St. Josefs-Krankenhaus Potsdam, Allee nach Sanssouci 7, 14471 Potsdam, Germany; 2 Neurocure Clinical Research Center, Charite-Universitätsmedizin Berlin, Berlin, Germany; 3 Department of Ophthalmology, Klinikum Ernst von Bergmann, Potsdam, Germany; 4 Institute of Biometry and Clinical Epidemiology, Charité-Universitätmedizin Berlin, Berlin, Germany; 5 Berlin Institute of Health, Berlin, Germany; 6 Department of Neurology, Oberhavel-Kliniken Hennigsdorf, Hennigsdorf, Germany; 7 Department of Neurology, University of California, Irvine, CA USA; 8 Department of Neurology, Charité-Universitätmedizin Berlin, Berlin, Germany; 9 Experimental and Clinical Research Center, Max Delbrueck Center for Molecular Medicine and Charité-Universitätmedizin Berlin, Corporate member of Freie Universität Berlin, Humboldt-Universität zu Berlin, Berlin, Germany

**Keywords:** Optic neuritis, Fingolimod, Interferon Beta-1b, Remyelination, Multifocal VEP

## Abstract

**Background:**

Neuroprotection and promotion of remyelination represent important therapeutic gaps in multiple sclerosis (MS). Acute optic neuritis (ON) is a frequent MS manifestation. Based on the presence and properties of sphingosine-1-phosphate receptors (S1PR) on astrocytes and oligodendrocytes, we hypothesized that remyelination can be enhanced by treatment with fingolimod, a S1PR modulator currently licensed for relapsing-remitting MS.

**Methods:**

MOVING was an investigator-driven, rater-blind, randomized clinical trial. Patients with acute unilateral ON, occurring as a clinically isolated syndrome or MS relapse, were randomized to 6 months of treatment with 0.5 mg oral fingolimod or subcutaneous IFN-β 1b 250 μg every other day. The change in multifocal visual evoked potential (mfVEP) latency of the qualifying eye was examined as the primary (month 6 vs. baseline) and secondary (months 3, 6 and 12 vs. baseline) outcome. In addition, full field visual evoked potentials, visual acuity, optical coherence tomography as well as clinical relapses and measures of disability, cerebral MRI, and self-reported visual quality of life were obtained for follow-up. The study was halted due to insufficient recruitment (*n* = 15), and available results are reported.

**Results:**

Per protocol analysis of the primary endpoint revealed a significantly larger reduction of mfVEP latency at 6 months compared to baseline with fingolimod treatment (*n* = 5; median decrease, 15.7 ms) than with IFN-β 1b treatment (*n* = 4; median increase, 8.15 ms) (*p* <  0.001 for interaction). Statistical significance was maintained in the secondary endpoint analysis. Descriptive results are reported for other endpoints.

**Conclusion:**

Preliminary results of the MOVING trial argue in support of a beneficial effect of fingolimod on optic nerve remyelination when compared to IFN-β treatment. Interpretation is limited by the small number of complete observations, an unexpected deterioration of the control group and a difference in baseline mfVEP latencies. The findings need to be confirmed in larger studies.

**Trial registration:**

The trial was registered as EUDRA-CT 2011–004787-30 on October 26, 2012 and as NCT01647880 on July 24, 2012.

## Background

Relapsing-remitting multiple sclerosis (RRMS) is the most common autoimmune disorder of the central nervous system (CNS) and the leading cause of acquired disability in young adults [1]. T-cell mediated autoimmunity directed against myelin components of the CNS is regarded as the central pathogenetic principle, leading to multifocal demyelination, oligodendrocyte apoptosis, and axonal dysfunction and transection [2]. Recovery from relapses involves remyelination of white matter and optic nerve lesions after recruitment and differentiation of oligodendrocyte precursors from the lesion perimeter [3, 4], but it is limited by axonal degeneration and glial scarring which are observed even at the earliest stages of the disease [5, 6]. With increasing age and longer MS duration, a neurodegenerative component may become predominant which progresses independent of inflammatory activity [7, 8].

In recent years, an increasing range of immunomodulatory and immunosuppressive treatments has become available which effectively reduce the frequency and severity of inflammatory attacks and the accumulation of relapse-related neurological deficits [9]. As an important therapeutic gap however, current disease-modifying therapies are not known to exert direct neuroprotective effects or enhance remyelination [10].

Optic neuritis (ON) represents one of the most frequent phenotypes of MS relapse and occurs as the first demyelinating event in about one out of 3 MS patients [11, 12]. ON offers an elegant opportunity for studies of remyelination and neuroprotection in MS [13]. In particular, optic nerve conduction can be followed up in a non-invasive, standardized manner by measuring visual evoked potentials (VEP): In the acute state, complete conduction block may occur in severely demyelinated sections of surviving axons, leading to reduction or loss of the VEP amplitude [14]. Within the first month, reorganization of ion channels enables continuous, non-saltatory conduction in demyelinated parts of the axons, and the degree of VEP delay corresponds to the expanse of the lesion [14]. Over the course of several months, remyelination re-establishes saltatory conduction which is reflected in a gradual decrease of the VEP latency [15, 16].

High-dose pulsed corticosteroid treatment of ON may shorten the duration of the acute attack but does not necessarily improve the long-term prognosis regarding visual function [17]. Intravenous methyl prednisolone, usually given at a dose of 1 g on 5 consecutive days, is frequently used for acute therapy, but dose-equivalent oral treatment may be equally effective [18]. While at 6 months, overall recovery of high-contrast visual acuity is usually good to excellent with 58% showing 20/20 vision and just 6% 20/50 vision or worse [17], more subtle impairment is found in many patients even after prolonged periods of time [19–21]. This may include reduced perception of color and contrast, or increased glare sensitivity when driving or working at computer screens, leading to a reduced quality of life [22]. VEP latencies remain prolonged even after full clinical recovery, indicating permanent loss or insufficient myelination of fast conducting axons of the optic nerve. Spectral domain optical coherence tomography (OCT) offers a reliable non-invasive technique to investigate changes in the thickness of retinal layers and their spatial distribution [23, 24]. Using OCT, irreversible loss of axons and retinal ganglion cell damage are detected as soon as four to 6 weeks after ON onset, being predictive of long-term visual outcome [25, 26]. These findings suggest a limited window of opportunity for effective neuroprotective therapy in ON. Currently however, no recommendations are available other than preventing further relapses by initiating a disease-modifying therapy as soon as diagnostic criteria of multiple sclerosis or high-risk clinically isolated syndrome (CIS) are met [27].

IFN-β 1b and IFN-β1a have been shown to decrease number of clinical relapses, development of new focal MRI lesions, and progression of disability in MS [28, 29]. Moreover, both forms of IFN-β significantly reduced the rate of conversion to clinically definite MS in patients with a first demyelinating event. Unlike other disease modifying therapies, they are licensed and recommended for use not only in RRMS, but also in CIS patients fulfilling MRI criteria [27]. The ability of IFN-β to reduce progression of disability as well as the interpretation of brain volume loss, especially in the first year of treatment, continue to be matters of debate [30, 31]. IFN-β is however not considered to have direct neuroprotective effects or enhance remyelination in MS patients.

Fingolimod binds to sphingosin-1 phosphate receptors (S1PR) [32], leading to aberrant phosphorylation, internalization and degradation of S1PR [33]. Since S1PR are required for lymphocytes to exit lymphatic follicular structures, fingolimod exerts immune modulation by sequestering pathogenic T- and B-cells from the blood stream [34]. Fingolimod is currently licensed for use in RRMS but not CIS. While a first-line option in the US and other countries, the European Medical Agency (EMA) label reserves fingolimod for second-line therapy. In addition to inducing S1PR degradation, S1PR-agonistic properties of fingolimod may be relevant to its biological activity [35]. S1PR are also present on neurons, astrocytes and oligodendrocytes as well as resident and CNS-invading myeloid cells, where they were shown to mediate neuroprotective and pro-regenerative effects in preclinical studies [36–49]. Fingolimod readily crosses the blood brain barrier [50]. Compared to placebo or IFN-β 1a, fingolimod reduced brain atrophy in MS patients which is thought to be driven by neurodegeneration and synaptic loss [51–53]. Hence, use of fingolimod in the context of acute autoimmune demyelination could offer an advantage in terms of enhanced remyelination, prevention of astrogliosis, and preservation of axons, even as an early treatment. The present study aimed to investigate protective and pro-regenerative effects of fingolimod on the optic nerve of patients recovering from acute unilateral optic neuritis.

## Methods

### Study design and participants

*MOdification of VIsual outcomes in optic Neuritis using Gilenya®* (MOVING) was an investigator-driven, randomized, rater-blind, active-controlled, parallel-group study which recruited patients from two German MS centers including one at the Charité university hospital in Berlin as well as two academic teaching hospitals acting conjointly as a study site in Potsdam. Eligible patients were 18 to 55 years of age with an acute first manifestation of unilateral optic neuritis in the qualifying eye with clinical onset within 30 days before screening. Additional requirements were an ipsilateral residual vision (Snellen) of at least 0.1 and an abnormal full-field VEP (ffVEP), elicited by 1° pattern reversal [54] with a P100 latency of at least 115 ms or a delay of at least 15 ms compared to the fellow eye. Moreover, participants had to fulfill diagnostic criteria of RRMS according to the 2010 McDonald criteria [55] or of CIS with at least two typical lesions on brain or spinal MRI. Patients were excluded if they had an Expanded Disability Status Scale (EDSS) [56] value greater than 6.0 or had suffered a demyelinating event other than ON within 30 days prior to screening. Apart from treatment of the acute ON with i.v. methylprednisolone, participants were required to have received either no disease-modifying treatment in the previous 3 months or to have been on stable immunomodulation using IFN-β or glatiramer acetate for at least 6 months. Premenopausal women were excluded if they were pregnant, breastfeeding or not using highly efficient contraception (Pearl index < 1). Patients were also excluded if according to the current EU labels and guidelines, treatment with IFN-β 1b or fingolimod, MRI studies or use of gadolinium contrast agent were contraindicated or if there was comorbidity with potential impact on the recovery from ON. A full list of inclusion and exclusion criteria is available in the Additional file 1.

### Randomisation and masking

Pre-specified randomization lists were generated for each study site by the central study pharmacy, stratified by residual vision (≤ 0.5 vs. > 0.5). Each allocation sequence used block permutation with a block size of 4. After a screening phase of up to 2 weeks, eligible patients were randomly assigned on a 1:1 basis to receive either a once-daily oral dose of 0.5 mg fingolimod or subcutaneous injections of 250 μg IFN-β 1b every other day for 6 months. All outcome-related data were collected by a blinded rating physician and blinded technicians. Participants and treating physicians were not blinded regarding the treatment arm but instructed to strictly maintain blinding of the raters. Patients who completed the core study had the option to participate in an additional follow-up visit after 12 months.

The intended sample size was calculated at 44 individuals per group based on previously reported data on mfVEP recovery in optic neuritis [15], an expected drop-out rate of up to 20% and the assumption of a 30% greater reduction of mfVEP latency after 6 months with fingolimod treatment compared to IFN-β 1b.

Following initiation of the study, additional treatment options for RRMS were licensed, including oral medications. In consequence, use of an injectable comparator made the study unattractive to many patients. Recruitment was significantly slowed and stopped prematurely at the request of the funding source, Novartis Pharma.

### Procedures

A treating neurologist at each site was responsible for assessing eligibility, obtaining informed consent and supervising study procedures in an unblinded manner, including drug treatment, safety assessments, validation of co-medications, and handling of adverse events. All outcome-related data were collected in a blinded manner by independent study personnel. The treatment period started on the day of randomization (baseline visit) by administering the first dose of study drug at the study site. For patients randomized to fingolimod, 6 h first dose cardiac monitoring was performed as mandated by the Summary of Product Characteristics, and an additional safety visit was scheduled 2 months after the last dose. In the IFN-β arm, participants were trained in self-injecting the drug, and site personnel were available for troubleshooting between scheduled visits if needed. Treatment adherence was verified by counting returned medication at each visit. Three additional clinical visits were performed after 1, 3 and 6 months. A synopsis of assessments and details of the protocols are available in Supplementary Table S1. Ophthalmological endpoints including multifocal and full-field VEP, high and low contrast visual acuity, OCT, and self-reported visual quality of life as well as the Multiple Sclerosis Functional Composite Measure (MSFC) [57] and EDSS were obtained at baseline and after 3 and 6 months. MRI was performed at baseline and after 6 months. Adverse events reported by the participant or study personnel were recorded throughout the study. Per protocol, patients with incident recurring ON in the qualifying eye were excluded from the study. Other criteria for discontinuation are available in the Additional file 1.

### Outcomes

The primary endpoint was the improvement of the mfVEP latency from the qualifying eye at 6 months compared to baseline, indicating the degree of remyelination and restitution of axonal function of the optic nerve. Measurements were performed in triplicate using a RETIscan device (Roland Consult, Brandenburg, Germany) and automatic postprocessing by mfPerimeter software (Roland Consult). Secondary endpoints were the improvement of the mfVEP latency from the qualifying eye at 3 and 12 months compared to baseline. Further exploratory endpoints included high and low contrast visual acuity after 3 and 6 months as determined by the number of correctly recognized letters on 100 and 2.5% contrast Sloan charts; retinal nerve fiber layer thickness (RNFLT), total macular volume (TMV) and ganglion cell and inner plexiform layer volume (GCIPLV) after 3 and 6 months as measured by spectral domain OCT (Spectralis, Heidelberg Engineering, Heidelberg, Germany) [58], EDSS and MSFC at 3 and 6 months, as well as self-reported visual quality of life after 3 and 6 months, using the composite score of the National Eye Institute Visual Functioning Questionnaire (NEI-VFQ39) [59]. On 3 Tesla brain MRI (TIM Trio, Siemens, Erlangen, Germany), changes in the number and volume of T2 hyperintense lesions at 6 months compared to baseline and the number of contrast-enhancing lesions on T1-weighted images after 6 months were determined. For participants in the optional extension, all outcomes were again evaluated 12 months after baseline. For standardization, all studies were performed using a single mfVEP device located in Potsdam as well as a single OCT device and a single MR scanner located in Berlin. Complete definitions and technical details of the assessments are available in the Additional file 1. Study oversight, on-site monitoring and source data verification were performed independently by the clinical trial facility at NeuroCure Clinical Research Center, Charité-Universitätsmedizin, Berlin.

### Statistical analysis

Results are reported as frequencies, or median with minimum and maximum, depending on the scale of the data (Table 1). Primary endpoint was the change in latency of the multifocal visual evoked potential (mfVEP) of the affected eye after 6 months of treatment compared to mfVEP latency at baseline. The mfVEP was modelled over time using non-parametric (rank-based) ANOVA-like analyses for longitudinal data in factorial settings, package ‘nparLD’, in R software [60] including an interaction between time and treatment arm as indicator for group differences in the mfVEP latency change. As secondary analyses, this was repeated including also data from 3 and 12 months when available. OCT and mfVEP results in the contralateral eye as well as the difference between ipsilateral and contralateral eyes have also been assessed. Further important secondary endpoints analyzed in the same fashion were the cumulative number of T2 lesions and the corresponding lesion volume measured at baseline and 6 months as well as the NEI-VFQ39 composite score. All other secondary endpoints are given with descriptive statistics only. Mann-Whitney U tests were used to examine differences in patient age at screening as well as time between symptom onset and randomization between the two treatment arms. To examine comparability of mfVEP and ffVEP, Spearman’s correlation coefficient on ranks was calculated for each time point. Statistical analyses were performed using R software version 3.4.1. All tests were 2-sided, and statistical significance was determined at an alpha level of 0.05. All *p*-values constitute exploratory data analyses without adjustment for multiple comparisons. A re-estimate of the required sample size for the primary endpoint was generated using nQuery Advisor 7.0 software (Statistical Solutions Ltd., Cork, Ireland), based on the observed difference in mean mfVEP latency change at month 6 vs. baseline and pooled standard deviation.

**Table 1:** Descriptive statistics

		Fingolimod	Missing	IFN-β 1b	Missing
Male / Female [n]		3 / 3	–	2 / 5	–
CIS / RRMS [n]		2 / 4	–	6 / 1	–
Age [years]		41 (28; 51)	–	51 (24; 54)	–
EDSS	Baseline	1.5 (0; 3)	–	1.5 (1; 2)	–
	3 months	1.5 (0; 3)	1	1 (1; 1)	4
	6 months	1.5 (0; 3)	1	1.25 (1; 1.5)	3
MSFC	Baseline	−0.10 (−0.87; 0.21)	–	0.18 (−1.27; 1)	–
	3 months	−0.07 (− 0.30; 0.19)	1	0.47 (− 1.39; 0.87)	2
	6 months	0.09 (− 0.27; 0.59)	2	0.08 (− 1.36; 0.64)	1
mfVEP Latency [ms]	Baseline	112.65 (107.1; 120.3)	–	95.8 (90.7; 108.1)	–
	3 months	105.05 (92.8; 120.3)	2	120.3 (106; 120.3)	4
	6 months	94.8 (92.8; 113.2)	1	109.1 (94.8; 120.3)	3
	Δ = 6 months - Baseline	−15.7 (−19.3; 6.1)	1	8.15 (3.1; 24.5)	3
ffVEP P100 [ms]	Baseline	129.7 (119.2; 148.5)	–	122.1 (113.6; 145)	–
	3 months	125 (108; 144.4)	3	113.18 (112.15; 114.2)	5
	6 months	119.2 (93.9; 143.2)	1	109.8 (97.15; 110.4)	4
RNFLT [μm]	Baseline	94 (79; 116)	1	96 (84; 124)	1
	3 months	88.5 (82; 112)	2	82 (77; 92)	3
	6 months	83 (76; 110)	1	80.5 (75; 88)	3
	Δ = 6 months - Baseline	−9 (−13; −6)	2	−6 (− 14; − 3)	4
GCIPLV [mm^3^]	Baseline	1.75 (1.46; 2.23)	1	1.7 (1.44; 2.13)	1
	3 months	1.79 (1.67; 2.14)	2	1.65 (1.42; 1.89)	3
	6 months	1.72 (1.45; 2.14)	1	1.68 (1.38; 1.89)	3
	Δ = 6 months - Baseline	−0.065 (−0.09; −0.01)	2	−0.03 (−0.16; 0.01)	4
TMV [mm^3^]	Baseline	8.33 (8.14; 9.34)	1	8.36 (6.58; 9)	1
	3 months	8.5 (8.12; 9.31)	2	8.52 (7.58; 8.71)	3
	6 months	8.25 (8.03; 9.06)	1	8.46 (7.5; 8.7)	3
	Δ = 6 months - Baseline	−0.28 (−0.3; −0.09)	2	−0.12 (− 0.41; − 0.11)	4
Sloan 100%	Baseline	32 (29; 39)	1	37 (35; 39)	5
[n letters]	3 months	35 (35; 39)	3	40 (40; 40)	6
	6 months	40 (35; 44)	1	39 (29; 40)	4
Sloan 2.5%	Baseline	0 (0; 9)	1	2.5 (0; 5)	5
[n letters]	3 months	4 (3; 5)	3	2 (2; 2)	6
	6 months	6 (0; 15)	1	1 (0; 13)	4
NEI-VFQ 39	Baseline	87.46 (76.63; 92.08)	3	88.71 (72.5; 94.28)	3
Composite Score	3 months	92.84 (81.40; 94.33)	3	87.52 (75; 97.05)	3
	6 months	90.15 (78.30; 98.58)	3	74.60 (71.44; 84.85)	3
T2 Lesion Count	Baseline	34 (21; 64)	–	33 (15; 56)	–
[n]	6 months	34 (23; 37)	1	31.5 (14; 54)	3
	Δ = 6 months - Baseline	2 (0; 2)	1	−1 (−2; 1)	3
T2 Lesion Volume	Baseline	3.57 (1.88; 5.5)	–	2.95 (0.91; 11.89)	–
[ml]	6 months	4.56 (2.11; 5.89)	1	2.77 (1.24; 12.56)	3
	Δ = 6 months - Baseline	0.362 (0.11; 0.47)	1	0.31 (0.09; 0.67)	3
T2 New Lesion Count [n]	6 months - Baseline	2 (0; 3)	1	1.5 (1; 4)	3
T2 New Lesion Volume [ml]	6 months - Baseline	0.11 (0.00; 1.234)	1	0.226 (0.119; 1.085)	3
T2 Lesions with Increased Volume [n]	6 months - Baseline	12 (4; 18)	1	12.5 (4; 18)	3
T1 Gd-enhancing	Baseline	0 (0; 3)	1	0 (0; 6)	2
Lesions [n]	6 months	0 (0; 1)	1	0 (0; 2)	3
	Δ = 6 months - Baseline	0 (0; 1)	2	0 (0; 0)	4

Results are reported as frequencies, or median and range. RFNLT, GCIPLV, TMV, mfVEP and ffVEP are reported for the affected eye

## Results

**Fig. 1:**
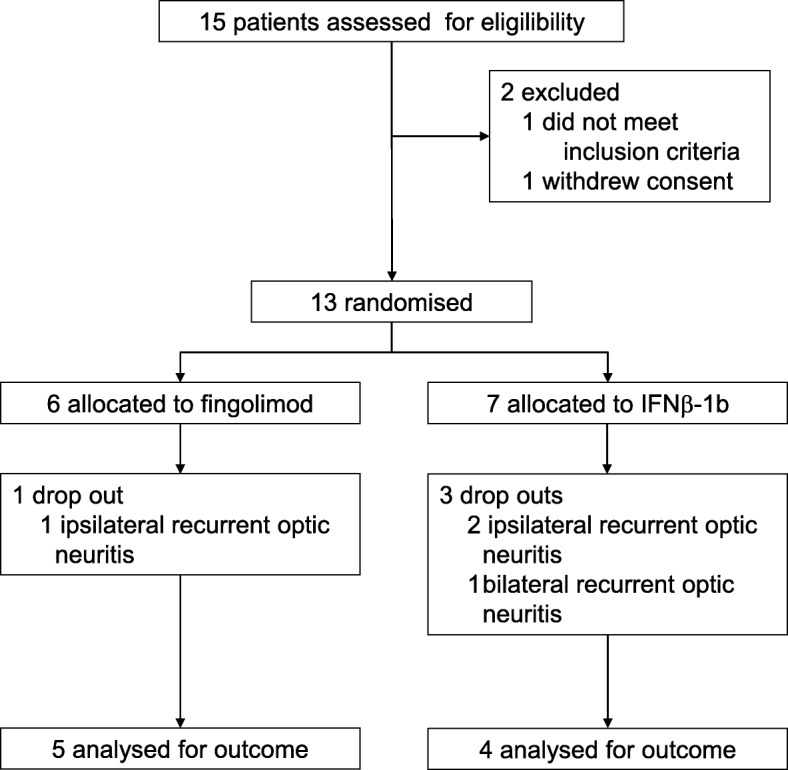
Patient disposition

Participants were enrolled between June 2013 and April 2015. Of 15 screened patients, 13 were eligible for inclusion (Fig. 1). One patient withdrew consent, and one patient suffered a relapse of optic neuritis while in the screening period. Of the eligible patients, 6 were randomized to fingolimod and 7 were randomized to INF-β 1b. Median delay between onset of symptoms and randomization was 27 days in the fingolimod arm (range, 22–37 days) and 33 days in the interferon arm (range, 16–49 days; *p* = 0.285; Mann-Whitney U test). All patients received their assigned treatment. A total of 9 participants completed the 6 months treatment period, and 6 of these were available for extended follow-up at month 12. Two participants in the fingolimod arm sustained relapses. One of these had recurrent ON in the qualifying eye, leading to exclusion from the study. In the interferon arm, two participants relapsed. Each had recurrent ON (ipsilateral, *n* = 2; bilateral *n* = 1), also leading to exclusion.

Descriptive statistics including ipsilateral OCT and VEP are given in Table 1. OCT and mfVEP results in the contralateral eye as well as the difference between ipsilateral and contralateral eyes can be found in the Additional file 1 (Supplementary Tables S2 and S3). Baseline characteristics revealed a higher frequency of CIS in the interferon group (6 out of 7) compared to the fingolimod group (2 out of 6). Furthermore, patients with fingolimod treatment had lower MSFC scores and visual acuity as well as longer median mfVEP and ffVEP latencies at baseline compared to the IFN-ß 1b arm. Age at screening was more widely distributed in the interferon group (24–54 years; median, 51 years; mean, 42.0 years) than in the fingolimod group (28–51 years; median, 41 years; mean, 39.7 years). No significant difference in age was detected between groups (*p* = 0.617; Mann-Whitney U test).

**Fig. 2:**
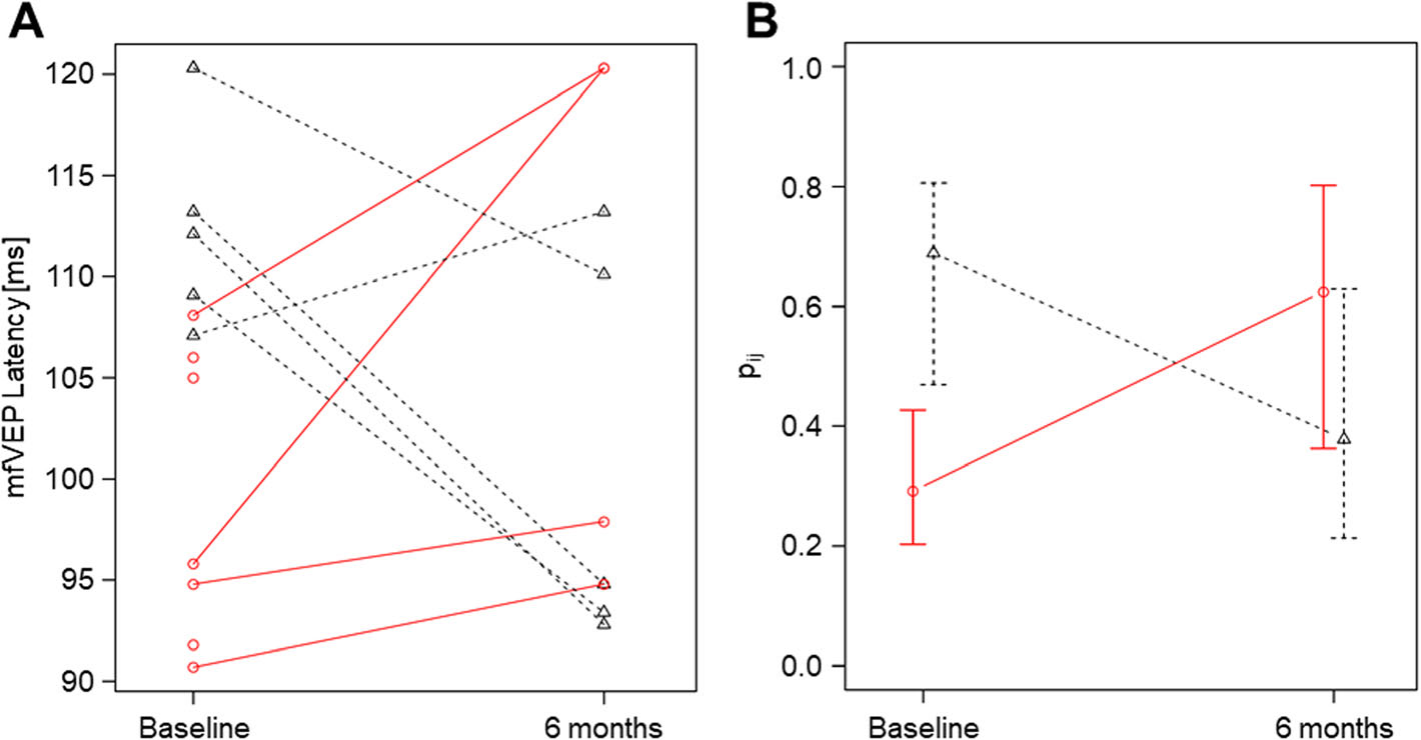
Treatment effect on change in multifocal VEP latency from the qualifying eye. Preliminary analysis of the primary endpoint, change in mfVEP latency from the qualifying eye. Black, fingolimod. Red, IFN-β 1b. **a** Individual follow-up of the mfVEP latency from the qualifying eye. **b** Results of the non-parametric longitudinal data analysis using the R package ‘nparLD’. Relative treatment effects with 95%-confidence intervals for complete cases only (fingolimod, *N* = 5; IFN-β 1b, *N* = 4; *p*- value for interaction, 0.001)

No eyes had loss of the mfVEP signal at screening or during follow up. Efficacy analysis of the primary endpoint including only complete measurements at baseline and 6 months follow-up showed that the latency of mfVEP from the affected eye increased compared to baseline in the IFN-β arm while it decreased over time in the fingolimod arm. Nonparametric modelling revealed a significant difference in change over time between the two study arms (interaction arm*time: *p* = 0.001, Fig. 2). Main effects of time and treatment arm were not statistically significant. Relative treatment effects with 95% confidence intervals are given in Table 2. Secondary analyses including also 3 months and 12 months data showed similar results, although there were only 3 complete cases per treatment arm (Table 2 and Supplementary Figure 1). Intention-to-treat evaluation of these endpoints is depicted in Supplementary Figure 2. In the contralateral eye, there was a similar median increase of the mfVEP latency in the interferon group and a smaller median decrease in the fingolimod arm, but the difference in change over time was not statistically significant (interaction arm*time: *p* = 0.410, Supplementary Figure S3 and Supplementary Table S2). Similar observations were made regarding the relative delay of the mfVEP latency from the affected eye compared to the contralateral eye (interaction arm*time: *p* = 0.269, Supplementary Figure S4 and Supplementary Table S2).

**Table 2:** Statistical analysis of endpoints

	Time	Relative Effect, Fingolimod	N	Relative Effect, IFN-β 1b	N	*P*-value Treatment Arm	*P*-value Time	*P*-value Interaction
mfVEP Latency	Baseline	0.6889 (0.4692; 0.8060)	5	0.2917 (0.2029; 0.4268)	4	*0.630*	*0.905*	*0.001*
	6 months	0.3778 (0.2134; 0.6298)	5	0.6250 (0.3622; 0.8020)	4			
T2 Lesion Count	Baseline	0.4500 (0.2635; 0.6684)	5	0.5139 (0.2871; 0.7316)	4	*0.935*	*0.239*	*0.164*
	6 months	0.5333 (0.3257; 0.7209)	5	0.5069 (0.2423; 0.7653)	4			
T2 Lesion Volume	Baseline	0.5056 (0.3180; 0.6902)	5	0.4167 (0.2184; 0.6738)	4	*0.696*	*< 0.001*	*0.876*
	6 months	0.5722 (0.3642; 0.7400)	5	0.4861 (0.2654; 0.7161)	4			
mfVEP Latency	Baseline	0.6736 (0.3338; 0.8699)	3	0.2569 (0.1119; 0.5675)	3	*0.400*	*0.773*	*0.004*
	3 months	0.2778 (0.1084; 0.6384)	3	0.7292 (0.5522; 0.8409)	3			
	6 months	0.4514 (0.1781; 0.7694)	3	0.6528 (0.2843; 0.8734)	3			
	12 months	0.3125 (0.1371; 0.6153)	3	0.6458 (0.4587; 0.7875)	3			

Statistical evaluation of the primary endpoint, mfVEP at 6 months compared to baseline, and of secondary endpoints (T2 lesion count and volume on brain MRI at 6 months vs. baseline; mfVEP latency at 3, 6 and 12 months vs. baseline). Estimation of relative effect size and unadjusted *p* values using non-parametric ANOVA-type statistics. Values in parentheses represent 95% confidence intervals. Only complete cases are included

The cumulative number of T2 lesions and the corresponding lesion volumes did not significantly differ in change over time (baseline, 6 months) between treatment arms (Table 2). Low values of RNFLT, GCIPLV and TMV were present in the affected eye in most participants at baseline, with further deterioration over time (Table 1). Only minor intraindividual changes of contralateral TMV, RNFLT and GCIPLV at 6 months vs. baseline were observed in both treatment arms (Supplementary Table S2). Relative to the contralateral eye, both treatment arms displayed a similar median loss of RNFLT and GCIPLV, while TMV loss appeared to be nominally smaller in the fingolimod arm (Supplementary Table S3).

An apparent trend for improved visual quality of life with fingolimod treatment did not meet statistical significance (interaction treatment arm*time: *p* = 0.122; Supplementary Figure S5). No clear correlation was discernible between ipsilateral mfVEP and ffVEP latencies at any time point (baseline: *r* = 0.230 (*n* = 13); 3 months: *r* = 0.447 (*n* = 5); 6 months: *r* = − 0.241 (*n* = 8); 12 months: *r* = 0.414 (*n* = 7)).

The primary outcome was used for a re-calculation of the required sample size. The mean change in mfVEP latency over 6 months was − 11.5 ms in the fingolimod arm and + 11.0 ms in the interferon arm, corresponding to a difference of 22.5 ms between groups with a pooled standard deviation of 10.2 ms. The nominal effect size (Cohen’s d) of treatment with fingolimod vs. IFN-β 1b was calculated at 2.2. Considering the small number of complete observations, even a more conservative approach using Cohen’s d = 1.0 predicts that a sample size of 20 per group would be sufficient to determine a differential effect of the two treatments with a 0,05 two-sided significance level at 80% power, using a Wilcoxon (Mann-Whitney) rank-sum test.

No new safety signals were detected in either treatment arm. In total, there were 10 adverse events (AE) in 4 participants receiving fingolimod and 10 AE in 4 patients in the interferon arm. A causal relationship was considered possible to fingolimod for 2 AE and to IFN-β 1b for one AE, while causality could not be determined for two AE experienced by one IFN-β 1b-treated patient. A full list of AE is available in Supplementary Table S4. All AE were of mild or moderate intensity. One patient in the fingolimod arm sustained a serious AE (SAE) requiring hospitalization which was deemed unrelated to the drug. None of the AE or SAE resulted in death, discontinuation or change of treatment.

## Discussion

The MOVING trial investigated whether recovery from unilateral optic neuritis in MS or CIS patients is improved by the S1PR modulator fingolimod compared to conventional treatment with IFN-β 1b. For the primary endpoint, a decrease of the ipsilateral mfVEP latency was observed after 6 months of fingolimod treatment, while an increase was observed with IFN-β 1b, and statistical significance was met for the effect of treatment over time. Interpretation of these results is limited since pre-planned sample sizes were not reached.

The statistical model revealed no significant main effect of the study arm on the primary outcome, arguing against a chance effect of randomization. Rather, the effect of treatment over time was significant both in the primary analysis and when including the secondary endpoints, i.e. mfVEP latencies at 3 and 12 months. Multivariable analyses were not undertaken due to the small number of observations. It should be noted that the most severe forms of ON are not represented in this study, since patients with residual vision of less than 0.1 or complete loss of VEP signal were not eligible.

The median improvement of mfVEP latencies in the fingolimod group is at the upper limit of what has previously been reported with this method in a cohort selected by good visual recovery [15]. A trend towards an increase of mfVEP latencies in the IFN-β group was not expected. However, preclinical findings do not argue against negative effects of IFN-β on remyelination. IFN-β inhibited the differentiation of oligodendrocyte precursors in mixed glial cultures [61]. In cuprizone-induced demyelination, IFNβ-deficient mice showed accelerated remyelination of the corpus callosum [62], and long-term IFN-β treatment of mice aggravated viral-induced demyelination [63]. Conversely, no effect of IFN-β on ffVEP latency recovery or RNFL thinning was reported in a 16-week open label study of patients with clinically isolated optic neuritis [64].

Looking at possible confounders, a larger proportion of participants with established MS vs. CIS as well as higher numbers of T2 lesions were randomized to the fingolimod arm, suggesting a longer duration of demyelinating disease in this group, which could negatively affect the capacity for remyelination [65]. Also, a more pronounced delay of ipsilateral mfVEP and ffVEP latencies and poorer performance on 100 and 2.5% contrast Sloan charts seemed to be present at baseline in the fingolimod arm, which could differentially affect change rates but also suggests a more severe manifestation and poorer a priori prognosis of ON in this group.

Relapses including recurrent optic neuritis occurred more frequently in the interferon arm compared to fingolimod. The finding is most likely explained by a differential treatment effect on the prevention of relapses [52, 66]. However, except for one relapse in the fingolimod arm, all were events of recurrent ON and led to exclusion from the study. Patients who contributed to the analysis had similar low frequencies of new or enlarging T2 lesions as well as Gd-enhancing lesions at 6 months. It is therefore unlikely that the difference in mfVEP latency change between groups is mediated by clinical or subclinical disease activity.

In the pivotal trials in RRMS, fingolimod significantly reduced brain atrophy and disability progression over 12 to 24 months when compared to placebo or IFN-β treatment [51, 52]. However, it is not clear to what degree these findings indicate direct neuroprotection by fingolimod or reflect downstream effects of reduced inflammatory activity: In a pooled analysis of the TRANSFORMS trial, the presence of gadolinium enhancing T1 lesions at baseline was the strongest predictor of brain atrophy [66]. On the other hand, a post hoc analysis of the FREEDOMS trial indicated independent effects of the reduction of relapses and of decreased brain volume loss on disability progression [67]. In primary progressive MS, fingolimod had no significant effect on disability progression or brain atrophy [68]. Difficulties in studying neuroprotection by fingolimod in clinical trials, even using advanced MRI techniques or cognition as a surrogate endpoint, have been pointed out in a recent review [69]. Similarly, an increase in macular volume on OCT has been reported in fingolimod-treated patients [70], which however are likely to reflect off-target effects rather than neuroprotection [71]. In view of this context, the results of the MOVING trial may represent the first indication of a superior effect of fingolimod on remyelination and preservation of axonal function compared to interferon treatment.

In both treatment arms, conventional ffVEP demonstrated a trend towards reduction of P100 latencies after 6 months, consistent with earlier studies [16, 72–74]. However, ffVEP and mfVEP were only weakly correlated at baseline and after 3 and 6 months of follow-up. Unlike ffVEP, which largely represents macular vision, mfVEP examines nerve conduction from the entire visual field including peripheral lesions. Previous studies have pointed out a superior sensitivity and specificity of mfVEP compared to ffVEP in ON [75]. Interestingly, the differential effect of the two treatments in MOVING appeared to be in parallel with changes of the self-reported visual quality of life.

As further parameters of integrity of the optic system, RNFLT, GCIPLV and TMV were followed up in the qualifying eye. All parameters showed considerable variance at baseline. In both treatment arms, a large proportion of participants had reduced RNFLT even before randomization and displayed a further decrease after 6 months. No obvious differential effect of the two treatments was detectable, as was the case for TMV and GCIPLV or when using the contralateral eye as a reference. Post-inflammatory retinal atrophy is an expected outcome after ON [76]. GCIPLV loss may start as early as 8 days after ON onset [77], and RNFL thinning has been reported as early as after 1 month, being predictive of progressive atrophy at month 6 [78]. Increased RNFLT due to edematous swelling, which may be present in the hyperacute state [79], was not observed. These findings may indicate that a window of up to 6 weeks between clinical onset and randomization in the MOVING trial may be too generous since a relevant degree of neuronal loss may already have occurred [26], thus limiting the impact of neuroprotective therapies. Indeed, other studies reporting protective effects of erythropoietin [80] or phenytoin [81] randomized patients within 10 and 14 days after ON onset, respectively. Recovery from ON has also been studied in the recent multicenter RENEW trial of opicinumab [82]. Opicinumab, a monoclonal antibody directed against LINGO-1, was hypothesized to enhance remyelination by directly promoting proliferation and differentiation of oligodendrocyte precursors. Notably, LINGO-1 blockade has no detectable immune modulatory effects [83]. While no significantly different effect of anti-LINGO-1 (*n* = 41) vs. placebo treatment (*n* = 41) on ffVEP latency decrease was observed in the intention-to-treat analysis, per-protocol analysis of 69 patients indicated potential efficacy of this approach after 32 weeks. No effect on GCIPLV decrease was detected, which could again be related to a mean delay of 24 days between ON onset and start of treatment. Based on this discrepancy the authors argued that therapeutic windows may be longer for remyelination compared to neuroprotection [82].

Additional possible confounders which were not accounted for in the MOVING protocol have since been described. MOVING had no specific rules in place to limit the delay between ON onset and initiation of corticosteroid therapy, which may affect visual recovery [84, 85]. Moreover, we did not evaluate focal lesions in the posterior visual pathways, which were recently described to be associated with RNFLT loss and delayed VEP [86, 87].

The MOVING trial was hampered by slow recruitment and incomplete follow-up. An effort to enlist additional sites was made but remained unsuccessful. For ethical reasons, a confirmed diagnosis of MS or CIS with at least 2 T2 hyperintense lesions on MRI was required for inclusion. As a major obstacle, eligible patients often viewed the use of an injectable interferon in the comparator arm as unattractive once dimethyl fumarate, teriflunomide and alemtuzumab had become available as alternative treatments for relapsing MS. This is reflected in a high proportion of CIS patients among the participants. Given the difficult recruitment, protocol deviations were tolerated as long as minimum treatment requirements were met and an unbiased evaluation of the primary outcome was expected.

## Conclusions

To summarize, available data from the prematurely stopped MOVING trial lends tentative support to the hypothesis that treatment with fingolimod may be associated with a better recovery from unilateral optic neuritis compared to treatment with IFN-β 1b. If corroborated, the findings argue in favor of the hypothesis that fingolimod may promote remyelination in autoimmune demyelinating disease. The observed treatment effect however should be interpreted with caution, as it may be attributable to a positive effect of fingolimod, a detrimental effect of interferon beta, both, or even an artifact due to the low sample size and incomplete follow up.

Current and previous experience underlines the role of ON as a particularly suited model to study remyelination in autoimmune CNS demyelinating disease, including pharmacological effects [13]. Use of mfVEP rather than ffVEP as a primary endpoint may offer advantages in terms of sensitivity and required group sizes. MOVING may be viewed as an encouragement for a repeat clinical trial. From our current experience, we recommend a multicenter study with a significantly shorter window for inclusion after onset of ON. An oral comparator should be used to facilitate recruitment and support a true double-blind design. To reduce baseline heterogeneity, mfVEP rather than ffVEP should be used as an inclusion criterion. A low sample size of 20 per arm may be sufficient using ipsilateral mfVEP latency decrease over 6 months as the primary outcome.

## Supplementary information

**Additional file 1.** Supplementary methods and results.

## Data Availability

The datasets used and/or analysed during the current study are available from the corresponding author on reasonable request.

## Abbreviations

AE: Adverse event
CIS: Clinically isolated syndrome
CNS: Central nervous system
EDSS: Expanded Disability Status Scale
ffVEP: full-field visual evoked potentials
GCIPLV: Ganglion cell and inner plexiform layer volume
IFN-β: Interferon beta
LINGO-1: Leucine rich repeat and immunoglobin-like domain-containing protein 1
mfVEP: multifocal visual evoked potentials
MRI: Magnetic resonance imaging
MS: Multiple sclerosis
MSFC: Multiple Sclerosis Functional Composite measure
NEI-VFQ39: National Eye Institute Visual Functioning Questionnaire
OCT: Optical coherence tomography
ON: Optic neuritis
RNFL: Retinal nerve fiber layer
RNFLT: retinal nerve fiber layer thickness
RRMS: Relapsing-remitting multiple sclerosis
S1PR: Sphingosine-1-phosphate receptor(s)
SAE: Serious adverse event
TMV: Total macular volume
VEP: Visual evoked potentials
