# Supplementary material for: Fingolimod after a first unilateral episode of acute optic neuritis (MOVING) – preliminary results from a randomized, rater-blind, active-controlled, phase 2 trial
Source: BMC Neurol. 2020 Mar 3;20:75. doi: 10.1186/s12883-020-01645-z (PMC7052969; doi:10.1186/s12883-020-01645-z)
Supplement: Supplementary file 1 — Additional file 1. Supplementary methods and results. [file 12883_2020_1645_MOESM1_ESM.docx]

**Fingolimod after a first unilateral episode of acute optic neuritis (MOVING) – Preliminary results from a randomized, rater-blind, active-controlled, phase 2 trial**

Christian Albert^1^, Janine Mikolajczak^2^, Anja Liekfeld^3^, Sophie K. Piper^4,5^, Michael Scheel^2^, Hanna G. Zimmermann^2^, Claus Nowak^4.,5^; Jan Dörr^2,6^; Judith Bellmann-Strobl^2^; Claudia Chien^2^; Alexander U. Brandt^2,7^; Friedemann Paul^2,5,8,9^; Olaf Hoffmann^1,2^

^1^Department of Neurology, Alexianer St. Josefs-Krankenhaus Potsdam; ^2^Neurocure Clinical Research Center, Charite-Universitätsmedizin Berlin;^3^Department of Ophthalmology, Klinikum Ernst von Bergmann, Potsdam; ^4^Institute of Biometry and Clinical Epidemiology, Charité-Universitätmedizin Berlin; ^5^Berlin Institute of Health; ^6^Department of Neurology, Oberhavel-Kliniken Hennigsdorf, ^7^Department of Neurology, University of California, Irvine, CA, USA, ^8^Department of Neurology, Charité-Universitätmedizin Berlin;^9^Experimental and Clinical Research Center, Max Delbrueck Center for Molecular Medicine and Charité-Universitätmedizin Berlin, corporate member of Freie Universität Berlin, Humboldt-Universität zu Berlin

## SUPPLEMENTARY MATERIAL

**SupplementaRY Methods**

**Study flow.** Screening of potential participants was required to begin within 30 days after clinical onset of ON in the qualifying eye. Rationale, treatment plan and procedures were explained by the study physician, and the printed patient information was handed out. After informed consent was obtained, inclusion and exclusion criteria were verified, and examinations required to determine eligibility were performed. The screening period was limited to a maximum of 14 days. At the baseline visit, subjects who fulfilled all inclusion criteria and none of the exclusion criteria and who had given their informed consent were randomized to one of the treatment arms. Each site used two separate randomization lists for participants with visual acuity of up to vs. above 0.5 which had been prepared by the central study pharmacy. Treatment was initiated on the day of randomization by applying the first dose of the study drug. For participants randomized to fingolimod, first dose cardiac monitoring for at least 6 h was observed as mandated by the SPC. Patients randomized to IFN-β 1b were monitored for 1h and received instructions on how to self-administer the drug. During the treatment period, three further visits were scheduled as outlined in Supplementary Table 1. All participants were offered an optional follow-up visit after 12 months. An additional visit was scheduled 2 months after the final dose of fingolimod for safety purposes.

**Inclusion and exclusion criteria.** For inclusion in the MOVING trial, a diagnosis of acute unilateral optic neuritis was required with clinical onset within 30 days before screening. A visual acuity of at least 20/200 (decimal, 0.1) had to be preserved in the qualifying eye. On ffVEP, P100 latency from the qualifying eye was required to be ≥ 115 ms and/or delayed by ≥ 15 ms relative to the fellow eye. Only patients aged 18 to 55 years at screening with a diagnosis of relapsing-remitting multiple sclerosis according to the 2010 revised McDonald criteria (Polman 2011) or of a clinically isolated syndrome (CIS) were eligible. Specifically, at least 2 T2 hyperintense lesions typical of MS had to be present on a brain or spinal cord MRI study obtained at any time, not better explained by a different etiology. Clinical disability was limited to an EDSS from 0 to 6.0. At the time of randomization, participants were required to have received either no disease modifying therapy for at least three months (pulsed treatment of the ON using up to 5 x 1 g methyl prednisolone was permitted) or to be on stable immune modulation with IFN-β or glatiramer acetate for at least six months. Specific rules were in place regarding prior treatment with other disease modifying drugs, but no such patients were referred for screening. For female participants, a negative pregnancy test (hCG urine dipstick) at screening and randomization was mandatory as well as use of highly effective contraception (Pearl index < 1), reliable heterosexual abstinence or sterilization of the exclusive heterosexual partner. Written informed consent was required from all participants, including the recording and evaluation of pseudonymized clinical information for the purposes of the trial. Patients were not eligible if an MS relapse other than ON had occurred within 30 days prior to screening or if symptoms were better explained by an etiology other than MS. Progressive forms of MS were also excluded. Further exclusion criteria were conditions preventing or interfering with MRI or other trial specific procedures and studies, including allergy, intolerance or other contraindications to gadolinium containing MRI contrast agents. Patients with contraindications to IFN-β 1b or fingolimod according to the Extavia® and Gilenya® European medical agency’s summary of product characteristics (SPC) were ineligible. Specifically, these included pre-existing immune deficiency, increased risk of opportunistic infections, severe active and chronic infections such as viral hepatitis or tuberculosis, presence of malignancies other than basal cell carcinoma of the skin, and hypersensitivity to the active or other ingredients. A clinical history of varicella or vaccination resulting in a protective antibody titer was required. Uncontrolled epilepsy, depression and suicidal ideation were also contraindications. Presence of second-degree atrioventricular (AV) block (Mobitz Type 2) or higher, sick sinus syndrome, sinoatrial block, a history of asystole, symptomatic bradycardia or recurrent syncope, prolonged QTc of > 470 ms in women or > 450 ms in men, coronary heart disease including angina or previous myocardial infarction, ischemic cardiomyopathy, congestive heart disease, cerebrovascular disease, uncontrolled arterial hypertension or severe sleep apnea were not allowed. Patients with clinically relevant hepatic (Child-Pugh grade C), renal or bone marrow dysfunction also were ineligible, e.g. defined by hemoglobin < 8.5 g/dL, white blood cell count < 2.5/nL, thrombocytes < 100/nL, creatinine clearance < 110mL in men or < 95mL/min in women (according to Cockroft and Gault formula; lower limit of normal decreases by 10mL/min per decade above 30 years), transaminases > 3.5 times the upper limit of normal, total bilirubin >2.0 mg/d, or evidence of monoclonal gammopathy. Patients were ineligible if they were participating in a different clinical trial, either ongoing or within three months prior to screening. Pregnant and breastfeeding women were excluded from the study. Medical, psychiatric and other conditions interfering with the ability to comprehend the patient information, give informed consent, comply with the study protocol or complete the study had to be ruled out. Specific to the study hypothesis, no comorbidities with potential impact on the recovery of visual function were allowed, such as diabetic retinopathy, glaucoma, uveitis or retinal detachment.

**Criteria for discontinuation.** Participants were informed that they could decline further participation in the study at any time, including withdrawal of consent. Participants were excluded from further treatment in the study if any of the above exclusion criteria became fulfilled, e.g. occurrence of pregnancy or medication-related safety issues. Medication was also to be discontinued if less than 75 % of the prescribed doses of IFN-β 1b were used, or if either more than 20 consecutive doses or more than 30 cumulative doses of fingolimod were missed. Other reasons for discontinuation included unauthorized use of the study medication, inclusion in another interventional trial, unblinding of the rating team e.g. in case of a medical emergency, loss of contact, or need for therapeutic interventions not authorized by the study protocol, e.g. switch to a different disease modifying therapy. In case of significant protocol violations, a decision to continue or discontinue was made by the treating physician and/or the leading investigator, based on their assessment of whether unbiased evaluation of the primary endpoint and hypothesis was still feasible. Patients who were discontinued from study treatment were asked to participate in the follow-up visits for intent-to-treat analyses. With respect to the primary endpoint, re-occurring ON in the qualifying eye resulted in exclusion from the study. Patients experiencing other MS relapses were allowed to remain in the study and continue the study medication after informed re-consent, and up to two courses of methyl prednisolone each including up to 5 doses of up to 2 g were permitted if required.

**Study Endpoints.** The primary endpoint was defined as the decrease in mfVEP latency measured in milliseconds from the qualifying eye after 6 months compared to baseline (for methodology see below). Secondary endpoints were the decrease in mfVEP after 3 and 12 months compared to baseline. Exploratory endpoints included ffVEP using standard clinical methodology as well as OCT and MRI parameters (see below). For exploratory parameters of vision, Sloan charts with 100 % contrast and 2.5 % contrast were presented at 4 m distance while covering the unaffected eye, and the number of correctly identified characters was counted. For all visual tests, participants were instructed to wear prescribed eyeglasses. EDSS and MSFC were obtained by qualified examiners. The self-administered NEI-VFQ39 questionnaire on visual quality of life was completed by the participants. All endpoints were obtained at 3 and 6 months as well as after 12 months in patients participating in the voluntary extension visit, and changes were compared to baseline values.

**mfVEP Protocol.** All measurements were performed using a single RETIscan device and mfPerimeter software (Roland Consult, Brandenburg an der Havel, Germany) by a specifically trained optometrist. For visual stimulation, a target-shaped checker board was displayed on a 21” computer screen. There were 58 sectors, each containing 8 black (< 1 cd/m^2^) and 8 white (at least 120 cd/m^2^) subsectors. The field diameter increased towards the periphery to address the cortical enlargement effect. Subjects were seated at a distance of 28 cm, resulting in a stimulated visual field of 54°. Studies were performed in a dark room. Prescribed reading glasses were to be worn, and the passive eye was covered. For registering visual evoked potentials, four active cup electrodes were placed on the scalp at a lateral distance of 5 cm from the inion as well as in the midline 4.5 cm above and 3.0 cm below the inion. A reference electrode was placed at the forehead. Abrasive electrode gel was used to achieve a scalp impedance of 5 kΩ or less. The recorded frequency range was set at 1 to 20 Hz. The software applied repeated stimulation to each sector in a pseudorandom sequence by contrast reversal of the subsectors. Four independent measurements were performed at each session. The software automatically detects the VEP latency from each segment, defined as the interval between stimulus onset and the highest positive deflection, usually the second peak of the VEP signal. For the primary endpoint of the study, the average latency was automatically calculated by the software. Segments without discernible cortical responses were not included in the average.

**MRI Protocol.** All MR studies were performed using a single 3 T device (Tim TRIO; Siemens, Erlangen, Germany). The MRI protocol included a volumetric high-resolution 3D T1 weighted sequence (TR/TE/TI = 1900/2.55/900 ms, FOV = 240 x 240 mm2, matrix 240 x 240, 176 slices, slice thickness 1 mm), a volumetric high-resolution 3D FLAIR sequence (TR/TE/TI=6000/388/2100 ms) and 3D T1 weighted sequence (TR/TE/TI = 1900/2.55/900 ms, FOV = 240 x 240 mm2, matrix 240 x 240, 176 slices, slice thickness 1 mm) after intravenous administration of gadobutrol (Gadovist®;

Bayer Vital, Leverkusen, Germany) at a dose of 0.1 ml/kg bodyweight. T2 hyperintense lesion were manually segmented on 3D FLAIR images using ITK-snap (Version 3.6.0.; www.itksnap.org) by an expert with 5 years of experience in lesion segmentation.

**OCT Protocol.** All measurements were obtained on a single spectral-domain OCT device (Heidelberg Spectralis, Heidelberg Engineering, Germany) using Heidelberg Eye Explorer viewing module version 5.6.1.0. RNFL thickness was derived from a 3.4 mm peripapillary ring scan (automatic real time, ART = 100). Ganglion cell and inner plexiform layer (GCIPL) volume and total macular volume (TMV) were extracted within a 6 mm cylinder centered on the fovea from a macular volume scan (25° x 30°, 61 vertical B-scans, high-speed mode, ART 12-15). OCT scans were checked for sufficient quality by an experienced rater and segmentation was manually corrected if necessary (P. Tewarie et al., PLoS One 2012; doi: 10.1371/journal.pone.0034823).

**Safety monitoring.** Vital signs including heart rate, systolic, diastolic blood pressure and body weight were recorded and a complete physical examination was performed at each visit. At baseline, a standard 12-channel electrocardiogram was obtained and evaluated by a qualified physician. In the fingolimod arm, a 6 h first dose cardiac monitoring was performed at the baseline visit, as specified in the current Gilenya® SPC. Adverse events (AE) and serious adverse events (SAE) were recorded at each visit. Hospitalization for the sole purpose of treating an MS relapse was not defined as a SAE. Laboratory studies including blood cell count, sodium, potassium, calcium, phosphate, creatinine, urea, aminotransferases and urine analysis were performed at each visit. In addition, leukocyte differential counts, c-reactive protein, thyroid stimulating hormone and immune fixation were included at baseline, and a hCG urine dipstick test was performed for women of childbearing age at all planned visits. For patients in the fingolimod arm, follow-up laboratory studies including blood count, leukocyte differentiation, c-reactive protein, creatinine and transaminases were scheduled two months after the last dose

**SupplementaRY Table S1: Schedule of visits and assessments**

|  | Screening | Baseline | Month 1^1^ | Month 3^1^ | Month 6^2^ | Safety^2,3^ | Month 12^4^ |
| --- | --- | --- | --- | --- | --- | --- | --- |
| Informed Consent | 🗸 |  |  |  |  |  |  |
| Inclusion and Exclusion Criteria | 🗸 | 🗸 |  |  |  |  |  |
| Pregnancy Test | 🗸 | 🗸 | 🗸 | 🗸 | 🗸 |  |  |
| Randomization |  | 🗸 |  |  |  |  |  |
| Relapses | 🗸 | 🗸 | 🗸 | 🗸 | 🗸 |  | 🗸 |
| Medical history | 🗸 | 🗸 | 🗸 | 🗸 | 🗸 |  | 🗸 |
| AE/SAE |  |  | 🗸 | 🗸 | 🗸 | 🗸 |  |
| Concomitant medication | 🗸 | 🗸 | 🗸 | 🗸 | 🗸 |  | 🗸 |
| Physical examination | 🗸 |  |  |  | 🗸 |  |  |
| Vital signs | 🗸 | 🗸 | 🗸 | 🗸 | 🗸 |  | 🗸 |
| Weight, height | 🗸 |  |  |  |  |  |  |
| EDSS, MSFC | 🗸 |  |  | 🗸 | 🗸 |  | 🗸 |
| ECG | 🗸 | 🗸 |  |  |  |  |  |
| Laboratory Studies | 🗸 |  | 🗸 | 🗸 | 🗸 | 🗸 |  |
| MRI | 🗸 |  |  |  | 🗸 |  | 🗸 |
| mfVEP | 🗸 |  |  | 🗸 | 🗸 |  | 🗸 |
| ffVEP | 🗸 |  |  | 🗸 | 🗸 |  | 🗸 |
| OCT | 🗸 |  |  | 🗸 | 🗸 |  | 🗸 |
| NEI-VFQ39 | 🗸 |  |  | 🗸 | 🗸 |  | 🗸 |
| Dispense study medication |  | 🗸 | 🗸 | 🗸 |  |  |  |
| Return and count unused medication |  |  | 🗸 | 🗸 | 🗸 |  |  |

^1^Time window ± 1 week; ^2^time window ± 2 weeks; ^3^applies to fingolimod arm only; to be scheduled 2 months after final dose. ^4^time window ± 4 weeks;

**SupplementaRY Table S2: mfVEP and OCT outcomes in the contralateral eye**

|  |  | **Fingolimod** | **Missing** | **IFN-β 1b** | **Missing** |
| --- | --- | --- | --- | --- | --- |
| **mfVEP Latency [ms]** | Baseline | 98.9 (94.8 ; 111.1) | 0 | 94.8 (91.8 ; 120.3) | 0 |
|  | 3 months | 110.1 (84.6 ; 120.3) | 2 | 103 (75.4 ; 115.2) | 4 |
|  | 6 months | 91.8 (87.7 ; 120.3) | 1 | 112.7 (97.9 ; 114.2) | 3 |
|  | Δ=6 months - Baseline | -6.2 (-23.4 ; 20.4) | 1 | 11.2 (-22.4 ; 21.4) | 3 |
| **RNFLT [µm]** | Baseline | 89 (74 ; 120) | 1 | 94.5 (78 ; 119) | 1 |
|  | 3 months | 93 (88 ; 121) | 2 | 85 (74 ; 91) | 3 |
|  | 6 months | 92 (82 ; 119) | 1 | 84.5 (74 ; 88) | 3 |
|  | Δ=6 months - Baseline | -0.5 (-1 ; 0) | 2 | 1 (-4 ; 2) | 4 |
| **GCIPLV [mm^3^]** | Baseline | 1.78 (1.55 ; 2.15) | 1 | 1.955 (1.76 ; 2.16) | 1 |
|  | 3 months | 1.945 (1.8 ; 2.16) | 2 | 1.885 (1.73 ; 2.04) | 3 |
|  | 6 months | 1.89 (1.58 ; 2.14) | 1 | 1.895 (1.73 ; 2.04) | 3 |
|  | Δ=6 months - Baseline | 0 (-0.05 ; 0.03) | 2 | 0.02 (-0.03 ; 0.07) | 4 |
| **TMV [mm^3^]** | Baseline | 8.32 (8.09 ; 9.07) | 1 | 8.575 (8.17 ; 9.28) | 1 |
|  | 3 months | 8.59 (8.32 ; 9.12) | 2 | 8.655 (8.23 ; 8.71) | 3 |
|  | 6 months | 8.36 (8.2 ; 8.93) | 1 | 8.62 (8.05 ; 8.7) | 3 |
|  | Δ=6 months - Baseline | -0.065 (-0.2 ; 0.04) | 2 | 0.02 (-0.12 ; 0.19) | 4 |

Results are reported as median and range**.**

**SupplementaRY Table S3: mfVEP and OCT outcomes relative to the contralateral eye**

|  |  | **Fingolimod** | **Missing** | **IFN-β 1b** | **Missing** |
| --- | --- | --- | --- | --- | --- |
| **mfVEP Latency [ms]** | Baseline | 10.7 (2.1 ; 25.5) | 0 | 1 (-29.6 ; 16.3) | 0 |
|  | 3 months | 4.1 (-18.3 ; 5.125) | 2 | 17.3 (-9.2 ; 44.9) | 4 |
|  | 6 months | 1 (-18.7 ; 22.5) | 1 | 2 (-16.3 ; 8.2) | 3 |
|  | Δ=6 months - Baseline | -2 (-30.6 ; 12.3) | 1 | 6.65 (-17.3, 26.5) | 3 |
| **RNFLT [µm]** | Baseline | 2 (-4 ; 5) | 1 | 3 (-3 ; 11) | 1 |
|  | 3 months | -6.5 (-10 ; -1) | 2 | 0.5 (-6 ; 3) | 3 |
|  | 6 months | -8 (-11 ; -5) | 1 | 0 (-8 ; 1) | 3 |
|  | Δ=6 months - Baseline | -8.5 (-13 ; -5) | 2 | -8 (-10 ; -4) | 4 |
| **GCIPLV [mm^3^]** | Baseline | -0.03 (-0.16 ; 0.08) | 1 | -0.175 (-0.72 ; 0) | 1 |
|  | 3 months | -0.14 (-0.17 ; -0.02) | 2 | -0.22 (-0.36 ; -0.13) | 3 |
|  | 6 months | -0.13 (-0.21 ; 0) | 1 | -0.21 (-0.42 ; -0.08) | 3 |
|  | Δ=6 months - Baseline | -0.06 (-0.08 ; -0.01) | 2 | -0.1 4(-0.18 ; 0.04) | 4 |
| **TMV [mm^3^]** | Baseline | 0,05 (-0,14 ; 0,27) | 1 | -0.02 (-2.7 ; 0.17) | 1 |
|  | 3 months | 0.05 (-0.28 ; 0.19) | 2 | -0.35 (-0.65 ; 0.05) | 3 |
|  | 6 months | -0.11 (-0.25 ; 0.13) | 1 | -0.18 (-0.55 ; 0.04) | 3 |
|  | Δ=6 months - Baseline | -0.12 (-0.32 ; -0.1) | 2 | -0.29 (-0.31 ; -0.13) | 4 |

Values are derived as affected minus contralateral eye. Results are reported as median and range**.**

**SUPPLEMENTARY TABLE S4: Adverse Events**

| **Treatment Arm** | **Adverse Event** | **Intensity** | **Relation to study drug** |
| --- | --- | --- | --- |
| Fingolimod | Upper respiratory infection | Mild | Unrelated |
|  | Abnormal liver function test | Mild | Possible |
|  | Bronchitis | Mild | Unrelated |
|  | Grade 1 AV block upon first dose | Mild | Possible |
|  | External otitis | Mild | Unrelated |
|  | Sore throat | Mild | Unrelated |
|  | Angina | Mild | Unrelated |
|  | Labial herpes | Mild | Unrelated |
|  | Steroid-induced acne | Mild | Unrelated |
|  | Muscular spasticity | Mild | Unrelated |
| IFN-β 1b | Rhinitis | Mild | Unrelated |
|  | Vitamin D deficiency | Mild | Unrelated |
|  | Vitamin D deficiency | Moderate | Unrelated |
|  | Arterial hypertension | Mild | Unrelated |
|  | Ocular pain | Mild | Unknown |
|  | Headache | Moderate | Unknown |
|  | Previously planned surgery | Mild | Unrelated |
|  | Flu-like symptoms with starting dose | Moderate | Possible |
|  | Viral enteritis | Mild | Unrelated |
|  | Common cold | Mild | Unrelated |

**SUPPLEMENTARY FIGURE S1**

**
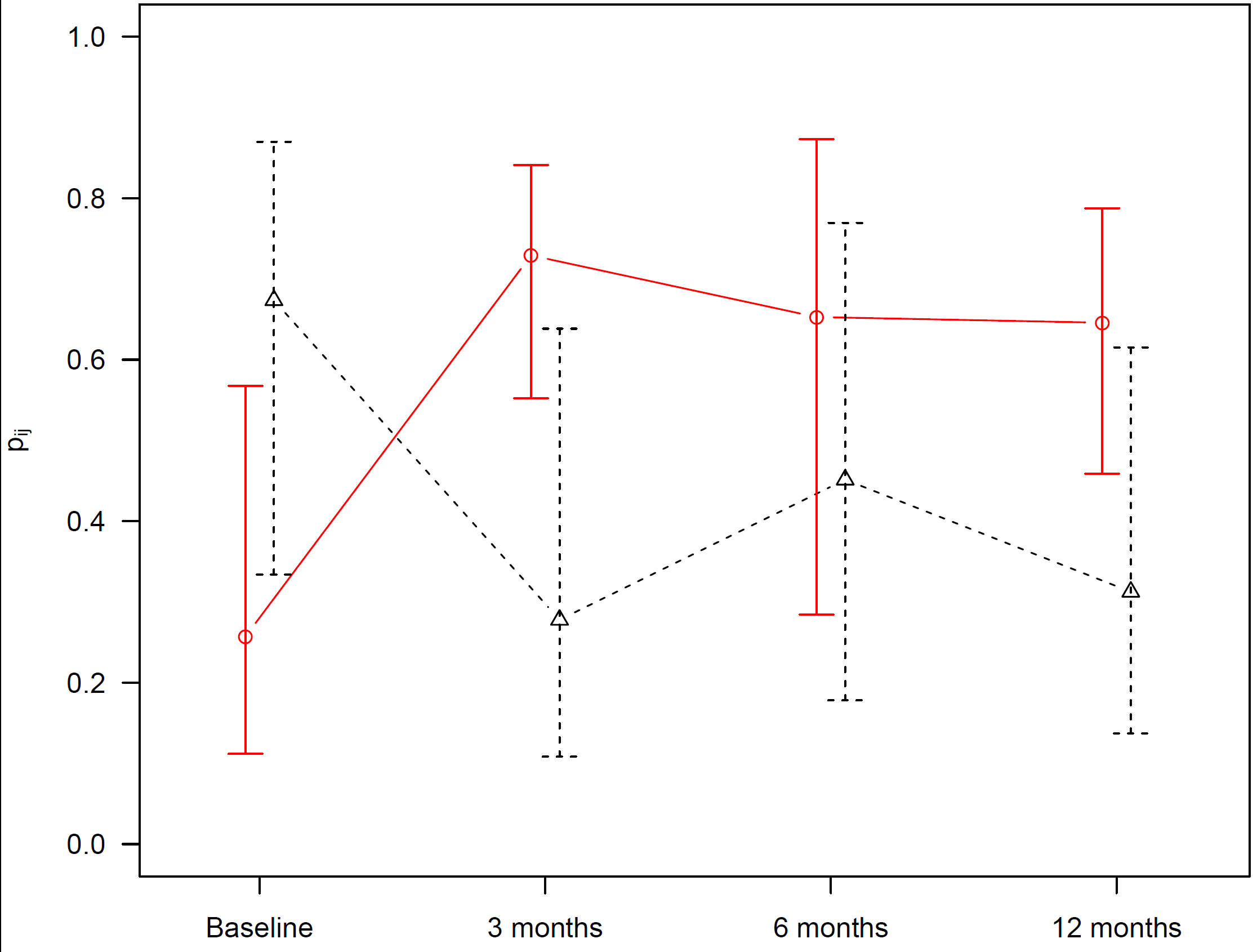
**

**Supplementary Figure S1.** mfVEP latency from the qualifying eye. Results of the non-parametric longitudinal data analysis using the R package ‘nparLD’, including 3 and 12 month data (secondary endpoint). Relative treatment effects with 95%-confidence intervals for complete cases only (p-value for interaction, 0.004). Black, fingolimod, N=3; Red, IFN-β 1b, N=3.

**SUPPLEMENTARY FIGURE S2**


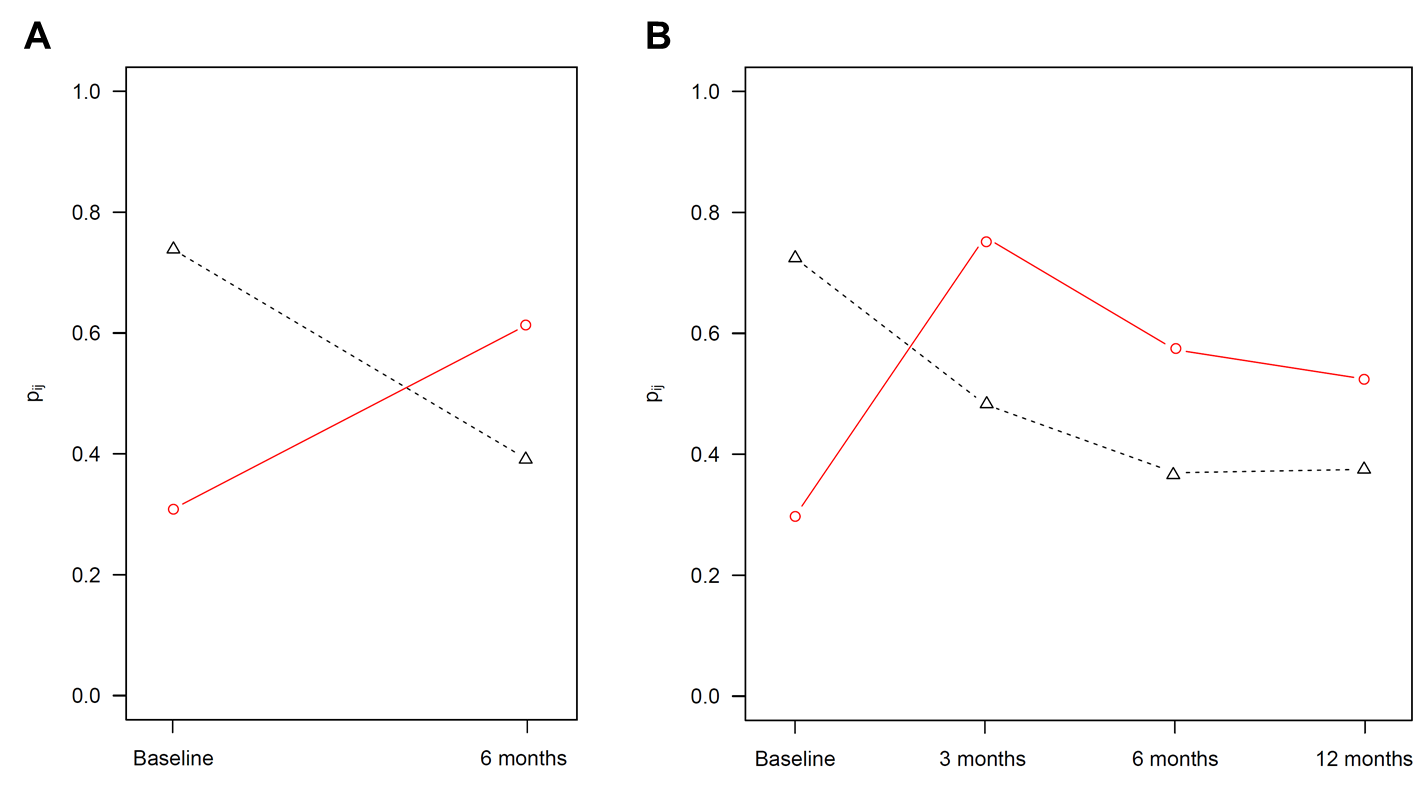


**Supplementary Figure S2.** Intention-to-treat analysis of the primary endpoint: mfVEP latency from the qualifying eye, non-parametric longitudinal exploratory analysis using the R package ‘nparLD’. Relative treatment effects including incomplete cases. Black, fingolimod, N = 6. Red, IFN-β 1b, N = 7. A) primary endpoint, mfVEP improvement over 6 months. B) secondary endpoint including 3 and 12 month data.

**SUPPLEMENTARY FIGURE S3**


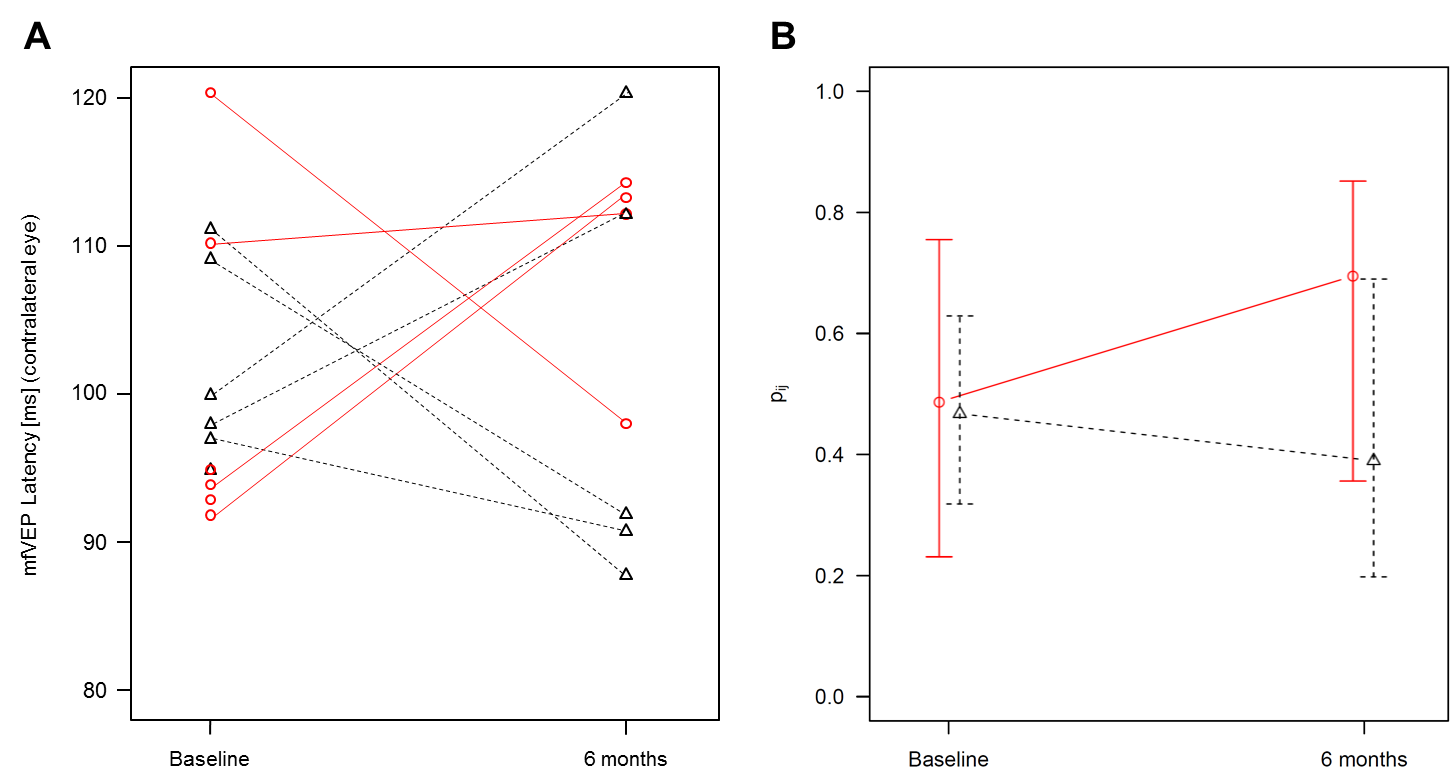


**Supplementary Figure S3.** Exploratory analysis of the mfVEP latency from the contralateral eye. Black, fingolimod. Red, IFN-β 1b. A) Individual follow-up. B**)** Results of the non-parametric longitudinal data analysis using the R package ‘nparLD’. Relative treatment effects with 95%-confidence intervals for complete cases only (fingolimod, N=5; IFN-β 1b, N=4; p-value for interaction, 0.410).

**SUPPLEMENTARY FIGURE S4**


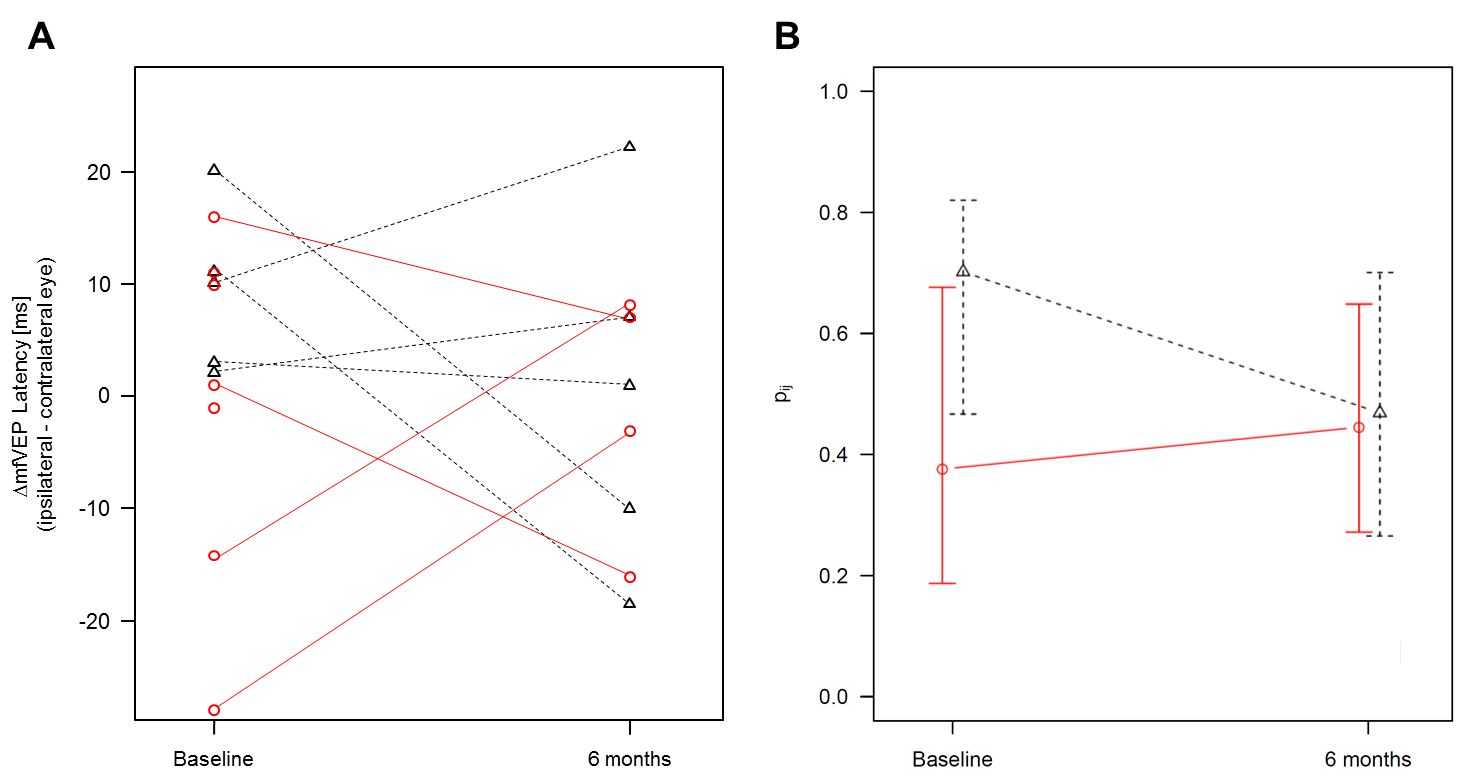


**Supplementary Figure S4.** Exploratory analysis of the interocular difference in mfVEP latencies, derived as ipsilateral latency minus contralateral latency. Black, fingolimod. Red, IFN-β 1b. A) Individual follow-up. B**)** Results of the non-parametric longitudinal data analysis using the R package ‘nparLD’. Relative treatment effects with 95%-confidence intervals for complete cases only (fingolimod, N=5; IFN-β 1b, N=4; p-value for interaction, 0.269).

**Supplementary Figure S5**


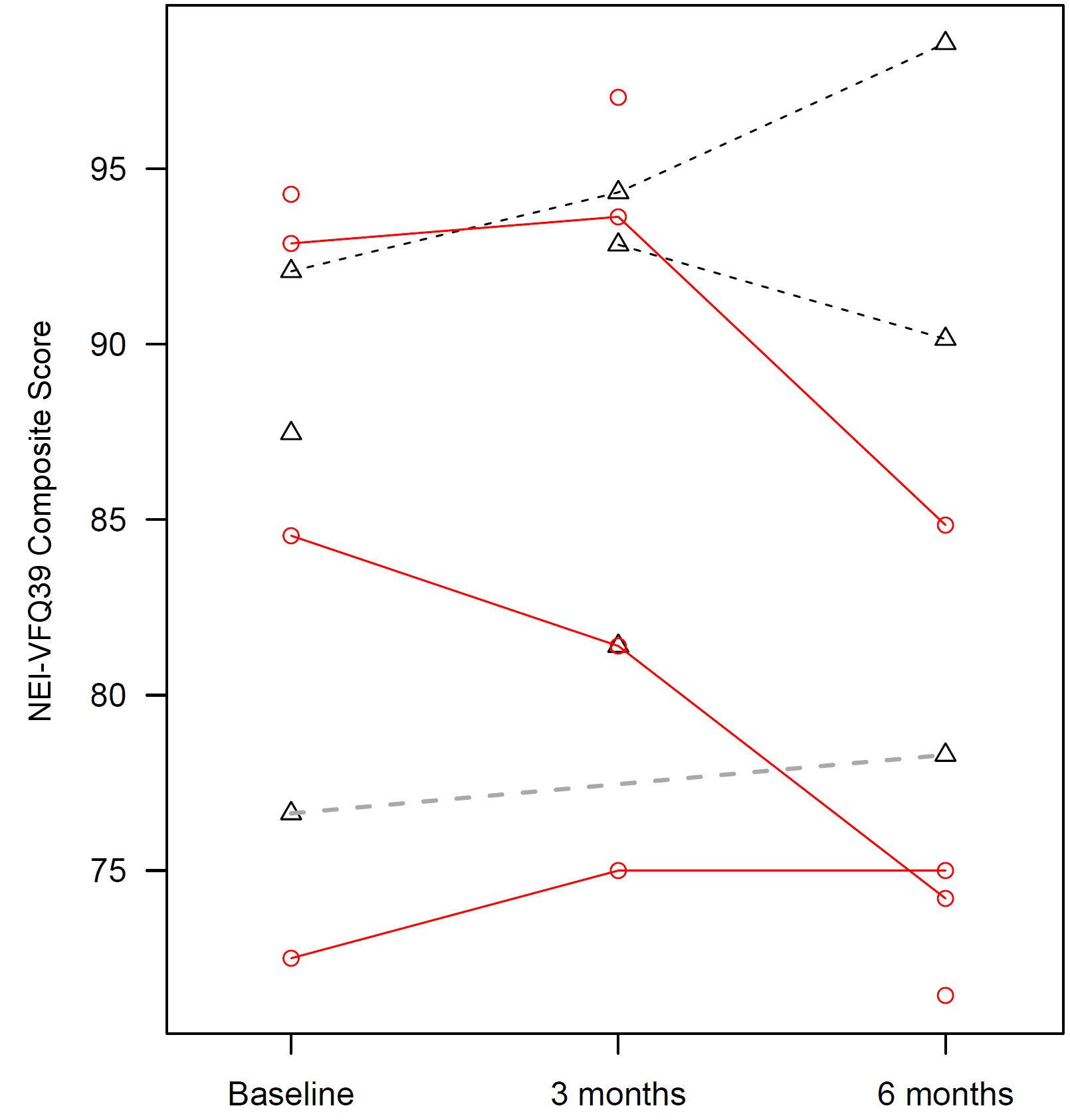


**Supplementary Figure S5.** Individual follow-up of the self-reported visual quality of life including incomplete cases. Black, fingolimod. Red, IFN-β 1b. Interaction treatment arm*time: p = 0.122
